# A new genomic resource dedicated to wood formation in *Eucalyptus*

**DOI:** 10.1186/1471-2229-9-36

**Published:** 2009-03-27

**Authors:** David Rengel, Hélène San Clemente, Florence Servant, Nathalie Ladouce, Etienne Paux, Patrick Wincker, Arnaud Couloux, Pierre Sivadon, Jacqueline Grima-Pettenati

**Affiliations:** 1 UMR CNRS/Université Toulouse III 5546, Pôle de Biotechnologies Végétales, 24 chemin de Borde Rouge, BP42617 Auzeville, 31326 Castanet Tolosan, France; 2 Current address : Syngenta Seeds SAS, BP27, 31790 Saint Sauveur, France; 3 Current address : INRA-UBP, UMR 1095, INRA Site de Crouël, 234 avenue du Brézet, 63100 Clermont-Ferrand, France; 4 Génoscope, CNRS, UMR 8030 and Université d'Evry, 91057 Evry, France; 5 Current address : Université de Pau et des Pays de l'Adour, UMR CNRS 5254 IPREM, IBEAS – BP1155, 64013 Pau Cedex, France

## Abstract

**Background:**

Renowned for their fast growth, valuable wood properties and wide adaptability, *Eucalyptus *species are amongst the most planted hardwoods in the world, yet they are still at the early stages of domestication because conventional breeding is slow and costly. Thus, there is huge potential for marker-assisted breeding programs to improve traits such as wood properties. To this end, the sequencing, analysis and annotation of a large collection of expressed sequences tags (ESTs) from genes involved in wood formation in *Eucalyptus *would provide a valuable resource.

**Results:**

We report here the normalization and sequencing of a cDNA library from developing *Eucalyptus *secondary xylem, as well as the construction and sequencing of two subtractive libraries (juvenile *versus *mature wood and *vice versa*). A total of 9,222 high quality sequences were collected from about 10,000 cDNA clones. The EST assembly generated a set of 3,857 wood-related unigenes including 2,461 contigs (Cg) and 1,396 singletons (Sg) that we named 'EUCAWOOD'. About 65% of the EUCAWOOD sequences produced matches with poplar, grapevine, *Arabidopsis *and rice protein sequence databases. BlastX searches of the Uniref100 protein database allowed us to allocate gene ontology (GO) and protein family terms to the EUCAWOOD unigenes. This annotation of the EUCAWOOD set revealed key functional categories involved in xylogenesis. For instance, 422 sequences matched various gene families involved in biosynthesis and assembly of primary and secondary cell walls. Interestingly, 141 sequences were annotated as transcription factors, some of them being orthologs of regulators known to be involved in xylogenesis. The EUCAWOOD dataset was also mined for genomic simple sequence repeat markers, yielding a total of 639 putative microsatellites. Finally, a publicly accessible database was created, supporting multiple queries on the EUCAWOOD dataset.

**Conclusion:**

In this work, we have identified a large set of wood-related *Eucalyptus *unigenes called EUCAWOOD, thus creating a valuable resource for functional genomics studies of wood formation and molecular breeding in this economically important genus. This set of publicly available annotated sequences will be instrumental for candidate gene approaches, custom array development and marker-assisted selection programs aimed at improving and modulating wood properties.

## Background

Wood is the major component of terrestrial plant biomass and is expected to play a significant role in future sustainable development as a renewable and environmentally acceptable source for fibers, solid wood and biofuel products [1,2]. Furthermore, wood is an important sink for atmospheric CO_2_, an excess of which is a major cause of global warming.

The production of wood or secondary xylem by xylogenesis is a remarkable example of terminal differentiation, producing a complex three-dimensional tissue specialized in conduction and mechanical support. This differentiation process comprises four major steps: cell division, cell expansion, deposition of lignified secondary cell wall and programmed cell death. The vascular cambium is the meristem tissue responsible for this differentiation process and, thus, for the extensive radial secondary growth of trees, ensuring regular renewal of functional secondary xylem and phloem during the lifespan of these perennial species.

Trees are long-living organisms that grow in a variable environment and are subject to developmental cues. As a consequence, wood is highly variable at the tissue level (in the proportions of different cell types) as well as at the cellular level (in cell size, shape, cell wall structure and composition). Anatomical, chemical and physical differences in wood properties are not only widespread from tree to tree, but also within a single tree [2]. For instance, variations between juvenile and mature wood present within the same tree produce distinct wood properties such as density and pulp yield [3].

The genus *Eucalyptus *is one of the main sources of wood worldwide and is the most widely used tree species in industrial plantations. Many *Eucalyptus *species are renowned for their fast growth, straight form, valuable wood properties, wide adaptability to soils and climates, and ease of management through coppicing [[4] and references therein]. According to the United Nations Food and Agriculture Organization [5], *Eucalyptus *is the principal hardwood species used for pulp extraction, with 19 million hectares of industrial plantations worldwide.

Because of their comparatively long generation times, forest trees are still at the early stages of domestication compared to crop species, with most breeding programs only one or two generations away from the wild. Nevertheless, the genetics of *Eucalyptus *is becoming one of the most advanced in forestry [4]. Nowadays, wood traits, which rely mainly on lignified secondary cell wall properties, are the key focus to many breeding programs. *Eucalyptus *breeding programs will thus benefit from genomic technologies that could significantly speed up the process of genetic improvement [4].

The genomes of most *Eucalyptus *species are very similar to those of poplar species, with a relatively small size (370–700 Mbp) and diploid inheritance (*n *= 11). In addition, the *Eucalyptus *trees are fast growing, most species are amenable to clonal propagation and some can be genetically transformed. These features make *Eucalyptus *particularly suitable for genomic technologies and a growing number of genetic tools (genetic, physical maps and quantitative trait loci) as well as EST collections are becoming available for some species. However, the huge commercial potential of eucalypts has fostered a situation in which access to genomic resources is restricted to a small number of private research consortia. These limitations may be overcome by the initiative of an International *Eucalyptus *Genome Consortium [6], which promoted the sequencing project of the *Eucalyptus grandis *genome undertaken by the US Department of Energy.

Because wood quality is a major trait that tree breeders would like to improve by using marker-assisted selection, it is important to increase publicly available *Eucalyptus *genomic resources, including putative candidate genes involved in the genetic control of wood properties. Indeed, recent advances in the molecular study of xylogenesis have revealed that wood formation is under strong genetic control, notably at the transcriptional level [7,8]. The production and analysis of ESTs from wood-forming tissues has increased our understanding of gene regulation involved in wood formation in tree species including loblolly pine [9-11], poplar [7,12], and white spruce [13]. Similarly, large scale sequencing of ESTs will be instrumental for the annotation of the *Eucalyptus *genome sequence. As a first step towards this goal, we have generated two secondary xylem subtractive libraries (xylem *versus *leaves and xylem *versus *phloem) rendering 487 unigenes preferentially or specifically expressed in differentiating secondary *Eucalyptus gunnii *secondary xylem [14,15], and providing a useful tool for gene profiling [16].

Here we present the sequencing of 9,216 normalized clones from a *E. gunnii *secondary xylem cDNA library generated in our laboratory [17]. In addition, we report the construction and sequencing of two suppression subtractive hybridization (SSH) libraries aimed at identifying genes differentially expressed in juvenile *vs *mature wood and *vice versa*. Sequencing of these EST libraries was performed in the framework of the French project FOREST [18] whose goal was to release ESTs sequences from woody species through public databases. *Eucalyptus *EST sequences produced in our lab have been assembled into a unigene dataset called EUCAWOOD and the unigenes have been functionally annotated and compared with other plant species. The functional annotation of the unigene set is discussed in the context of the wood formation process.

## Results and Discussion

### Construction and sequencing of normalized libraries

With the aim of sequencing a large number of ESTs representative of the set of mRNAs expressed in secondary xylem, we chose a cDNA library prepared from the differentiating secondary xylem of *E. gunnii *[Xyl_cDNA_] containing 1.5 × 10^6 ^clones [17], which has already proven a good source of genes expressed during wood formation [17-23]. Because in cDNA libraries, each cDNA occurs at a frequency proportional to that of its corresponding mRNA in the tissue it was prepared from, prevalent and intermediate frequency classes of mRNAs are expected to be overwhelming in a random large scale sequencing program. In order to minimize this redundancy and increase the chance of identifying low-expressed genes, we decided to normalize the Xyl_cDNA _library according to the protocol of Bonaldo [24]. During the normalization procedure, human *desmin *cDNA was added at 1,000 copies to the non-normalized library whereas *EgCAD2*, of which 31 cDNA copies were present before normalization, served as an internal control. After normalization, six copies of *desmin *and five copies of *EgCAD2 *were recovered, demonstrating that redundancy in the library was drastically reduced by the normalization procedure. Thus, the representation of the different genes expressed in secondary xylem was expected to be increased among the 9,216 clones of the normalized Xyl_cDNA _library as compared to the original library.

All 9,216 Xyl_cDNA _clones were sequenced from the 5' end. Following vector and low-quality sequence trimming, 8,043 high quality sequences with an average length of 566 nucleotides (nt) were retained. Sixty three percent (5,060) of the sequences were longer than 500 nt and only six percent (486) were shorter than 200 nt, indicating the quality of the library. These 8,043 sequences were deposited in the EMBL-EBI nucleotide database [EMBL: CT980028 to CT988078].

To complement this set of ESTs, we decided to seek for genes that are differentially expressed in juvenile and mature secondary xylem tissues. The transition from juvenile to mature xylem is known to be an important source of variation in wood quality [3]. We took advantage of SSH technology, known to equalize the level of representation of rare and abundant fragments [25], to reciprocally subtract cDNAs prepared from juvenile and mature secondary xylem tissues. Thus, we produced two SSH libraries: a juvenile *vs *mature (Jm) and a mature *vs *juvenile (Mj) secondary xylem library. Altogether, 818 clones were obtained and sequenced from both sides of the cloning site. A total of 1,179 good quality sequences with an average length of 412 nt were obtained, 604 from the Jm library and 575 from the Mj library. The sequences were deposited in the EMBL-EBI nucleotide database [EMBL: CT988079 to CT989251].

### EST assembly

**Figure 1:**
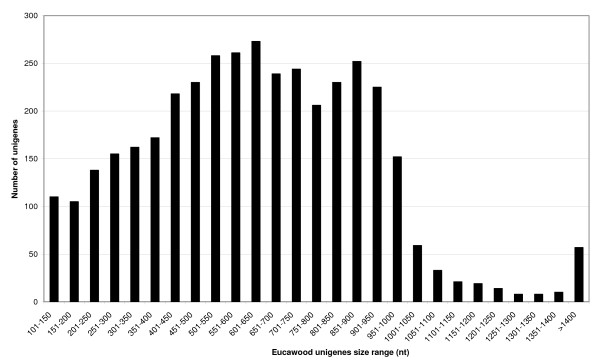
**Size distribution of the EUCAWOOD unigenes after assembly**.

The assembly of the 9,222 good quality sequences described above together with the ESTs and core nucleotide sequences publicly available in the GenBank and EMBL databases, generated 17,087 unigenes, comprising 7,921 contigs and 9,166 singletons. Among these, we discarded all sequences whose size was below 100 nt and selected for further analysis only the 3,857 unigenes (2,461 Cg and 1,396 Sg) which contained at least one sequence originating from one of our libraries including two SSH libraries previously obtained in the laboratory, *i.e*. a secondary xylem *vs *secondary phloem SSH library (Xp) [14] and a secondary xylem *vs *leaves SSH library (Xl) [15]. The rationale for this was to select a subset of secondary xylem-related sequences that we called 'EUCAWOOD' (see Additional file 1). The EUCAWOOD unigenes had an average length of 640 nt and a size distribution as shown in Figure 1. To mine this new *Eucalyptus *genome resource, we have developed a publicly accessible database that supports multiple queries on the EUCAWOOD unigenes and their functional annotation [26].

**Figure 2:**
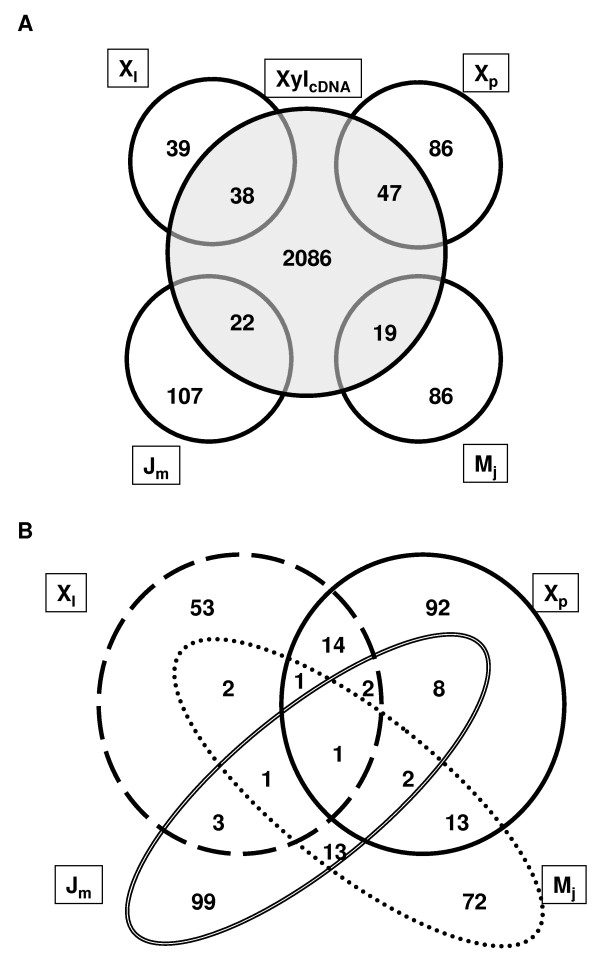
**Overlap between the EUCAWOOD unigenes**. (A) Venn diagram showing the overlap between unigenes originating from the cDNA library [Xyl_cDNA_] and each of the SSH libraries [Jm: juvenile *vs *mature secondary xylem; Mj: mature *vs *juvenile secondary xylem; Xl: secondary xylem *vs *leaves; Xp: secondary xylem *vs *secondary phloem]. (B) Venn diagram showing the overlap of unigenes derived from the four different SSH libraries.

The Venn diagram in Figure 2A illustrates the number of unigenes shared between the cDNA library (Xyl_cDNA_) and each of the four different SSH libraries. Interestingly, most of the contigs containing sequences originating from at least one of the SSH libraries (*i.e. *269) were not present in the Xyl_cDNA _library. Only 107 contigs contained ESTs originating from the Xyl_cDNA _and one of the SSH libraries (Figure 2A). This little overlap confirms the utility of combining cDNA and SSH libraries to identify new genes expressed in *Eucalyptus *secondary xylem: the SSH libraries contain many clones not recovered from the total cDNA library.

Figure 2B illustrates the low number of overlapping sequences between the four different SSH libraries. For instance, the Jm and the Mj subtractive libraries we generated assembled into 279 unigenes of which only 17 contained ESTs from both libraries. This limited overlap (6%) between the two libraries illustrates the efficacy of the subtraction procedure in the SSH technique. Most interestingly, the low overlap between the four libraries demonstrates the advantage of using several subtractive libraries to recover new genes distinct to each tissue.

### Sequence comparisons with other species

**Figure 3:**
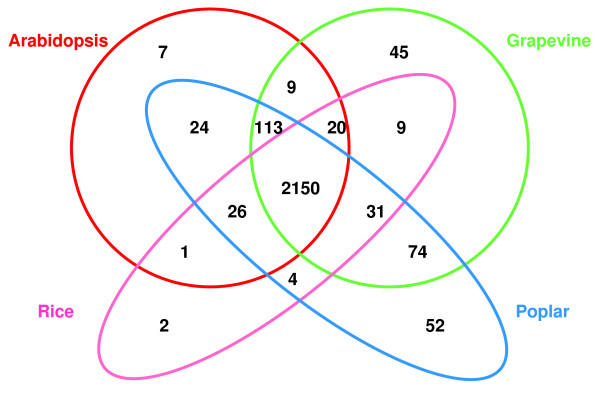
**Number of EUCAWOOD unigenes with similarities to predicted proteins from four plant species**. BLASTX searches (E value ≤ e^-10^) were conducted to identify EUCAWOOD unigenes in the JGI Poplar Proteins v*1.1*, *Arabidopsis TAIR7 Peptides, TIGR Rice Genome Annotation *and NCBI *(Vitis vinifera*) databases.

Homology searches were conducted using the BlastX program [27] to compare the "EUCAWOOD" unigene set with predicted protein and gene model databases for arabidopsis, poplar and rice, four plant species whose genomes have been sequenced [28-31]. These homology searches allowed us to assess the overlap between the EUCAWOOD unigenes and the protein sequence databases of these three model plants (Figure 3). Approximately 55% of the unigenes (2,150) matched sequences occurring in all three species and 65% (2,567) matched sequences in at least one of these three species. The highest number of hits were obtained with the two woody angiosperms, *i.e. *poplar (2,474) and grapevine (2,451), followed by arabidopsis (2,350) and rice (2,243) predicted protein sequences. Interestingly, 171 unigenes matched only against poplar and/or grapevine sequences, the only woody species whose genomes have been sequenced so far (see Additional file 2). Most of these 171 unigenes corresponded to unknown proteins, 45 of them only matched predicted proteins of *Vitis vinifera*, 52 had no other hit than gene models from poplar at an E-value cut-off of 10^-10^, 74 were common to poplar and grapevine. Further investigation is needed to verify whether these latter sequences correspond to genes specifically expressed during wood formation in trees.

### Functional annotation

**Figure 4:**
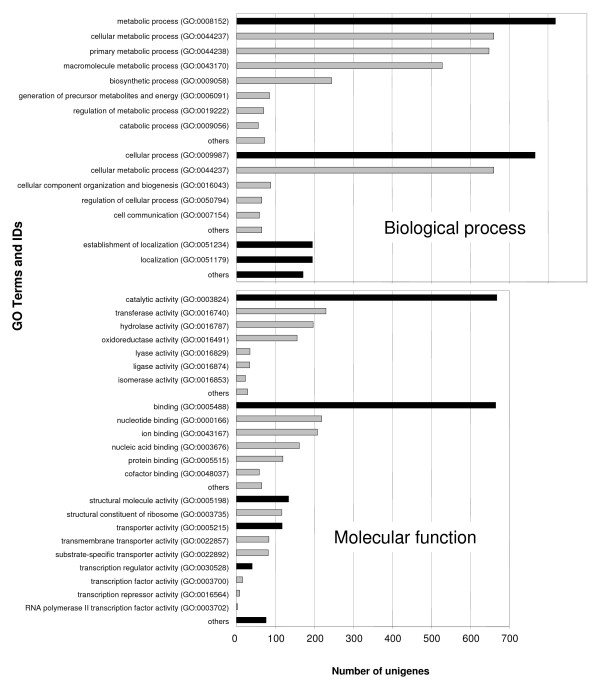
**Gene ontology assignments to EUCAWOOD unigenes**. GO terms were allocated to EUCAWOOD unigenes according to their best hit in searches of the Uniref100 database (E value ≤ e^-10^). Terms and IDs belonging to the 'Biological Process' and 'Molecular Function' categories are shown. Black bars indicate the main subcategories whereas the grey bars immediately below them illustrate subcategories therein. (Terms and IDs belonging to 'Cellular Component' category can be found in Additional file 1.)

To further allocate protein annotations to the EUCAWOOD unigenes, BlastX searches were performed against the Uniref100 database [32]. GO terms [33] associated with the best Uniref100 hit were then automatically assigned to the corresponding EUCAWOOD unigenes. Functional annotation data are presented in Additional file 1 as well as in the public EUCAWOOD database [26]. Overall, 2,466 (64%) unigenes produced matches to proteins in Uniref100. A total of 2,850 GO terms were allocated to 1,316 unigenes, filed under 'Biological Process' (1,018 terms), 'Molecular Function' (1,138 terms) and 'Cellular Component' (694 terms) (Figure 4 and Additional file 3). The vast majority of the 1,018 GO terms allocated to Biological Process genes fell under the categories 'Metabolism' (819 terms) and 'Cellular process' (767 terms) (Figure 4). The large proportion of unigenes involved in metabolic and biosynthetic processes confirms that differentiating secondary xylem is a very active tissue with a high metabolic rate. A large number of the terms allocated to 'Molecular Function' were in genes in the subcategories 'Catalytic Activity' (668 terms) and 'Binding' (665 terms) (Figure 4). The most represented activities in Catalytic Activity were transferases (230 terms), hydrolases (197 terms) and oxidoreductases (156 terms). The most abundant Binding activites were nucleotide binding (219 terms), iron binding (208 terms), nucleic acid binding (161 terms) and protein binding (119 terms).

**Figure 5:**
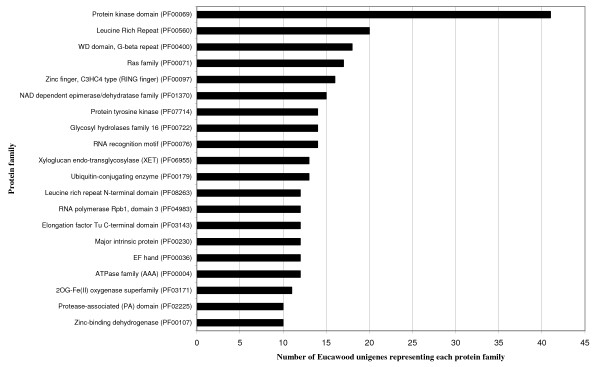
**Protein families among EUCAWOOD unigenes**. A total of 825 protein families from the PFAM protein family database were represented in the EUCAWOOD dataset. The black bars indicate the occurrence of the 20 most abundant protein families.

In a parallel annotation approach, we related the best Uniref100 hit of every unigene to the PFAM database [34,35] in order to identify protein families and domains in the EUCAWOOD unigene set. A total of 1,453 unigenes (37%) were assigned at least one PFAM identifier (ID) and, overall, 825 PFAM protein families and domains were represented among EUCAWOOD unigenes. Remarkably, PFAM IDs related to signal transduction and cell wall metabolism formed the majority of the 20 most abundant protein families (Figure 5 and Additional file 4). These PFAM matches showed that the most abundant protein families in the EUCAWOOD unigene set were also among the most represented in comparable studies with other plant species [13,36]. Similar examination of the various protein families represented in the subtractive libraries (Jm and Mj) revealed a completely different pattern from that of the EUCAWOOD dataset, in which the large majority of the unigenes originate from the Xyl_cDNA _library (Additional file 4). EUCAWOOD unigenes containing ESTs from Jm or Mj libraries produced matches with 57 and 47 different protein families, respectively, including only five families common to both Jm and Mj libraries. Among these 99 protein families, only seven appeared among the 20 most abundant families in the EUCAWOOD dataset. The PFAM annotation of the Uniref100 matches confirmed the little overlap between both libraries at the protein family level with only five common PFAM IDs.

Finally, 1,261 (32,7%) of EUCAWOOD unigenes produced no match against Uniref100, arabidopsis, poplar, grapevine or rice proteinsand were therefore considered as 'No Hits' at E value ≤ e^-10 ^(Additional file 5). The average length of the "No Hits" was remarkably shorter than that of the unigenes showing at least one BLASTX hit (447 nt *vs *738 nt). Consistent with this, the percentage of unigenes shorter than 400 nt was much higher among the 'No Hits' than among the 'Hits' (47.6% *vs *10.1%). The opposite was also true for unigenes longer than 800 nt: the percentage of unigenes longer than 800 nt was much lower among the "No Hits" than among the "Hits" (12% *vs *36%). The "No Hits" group is enriched in 3' sequences, which are usually less conserved than those upstream in the gene.

### Cell wall-related genes

One of the crucial stages in xylem differentiation is the formation of the secondary cell wall, which is largely composed of cellulose, lignin and hemicelluloses together with other less abundant polysaccharides and structural proteins [37]. We therefore mined the EUCAWOOD unigene set for genes involved in lignin biosynthesis, carbohydrate and cell wall metabolism. We performed BlastX searches using both the Cell Wall Navigator (CWN) [38,39], and the MAIZEWALL databases [40]. Altogether, 422 EUCAWOOD unigenes matched cell wall-related genes, with 142 and 380 hits with CWN and MAIZEWALL, respectively (Additional file 6). Among those, 101 were common to both databases, and 279 were found only in MAIZEWALL representing altogether the totality of the 18 categories described in this database. Most of the hits found only in MAIZEWALL were secondary cell wall-related genes including phenylpropanoid and lignin biosynthetic genes.

#### Lignin biosynthesis genes

All the gene families involved in the monolignol biosynthesis pathway were represented in the EUCAWOOD dataset including 18 unigenes (Additional file 7) with similarities to the set of lignin biosynthetic genes identified in *Arabidopsis *by Raes *et al *[41]. The EUCAWOOD set contained three distinct genes encoding hydroxycinnamoyl-CoA:shikinimate/quinate hydroxycinnamoyltransferase (*HCT*) suggesting that HCT in *Eucalyptus *is encoded by a small gene family as in poplar [42] rather than a single *HCT *gene, as in *Arabidopsis *[41]. Interestingly, eight ATP-binding cassette (ABC) transporters were present among EUCAWOOD unigenes, which might be involved in the transport of the lignin monomers to the cell wall through direct membrane pumping [43]. The molecular mechanism by which monolignols are incorporated into the lignin polymer is thought to involve key oxidation steps catalyzed by laccases and peroxidases [44]. Six putative laccases were found among the EUCAWOOD unigenes, one of which was most similar to *TT10/AtLAC15*, which has recently been proven to play a role in lignin synthesis [45]. Three of these six unigenes were similar to *IRX12/LAC4*, a gene involved in cell wall biosynthesis [46]. The expression of *IRX12/LAC4 *might be regulated by *At*MYB26/MALE STERILE34, a MYB transcription factor involved in secondary thickening of the anthers in *Arabidopsis *[47]. Eight EUCAWOOD unigenes were annotated as encoding peroxidases. Three of them are homologues of *At*PER12 and *At*PER64, two proteins whose precise biochemical functions remain elusive but which have been located in the cell wall [48].

#### Carbohydrate active enzymes and cell wall metabolism genes

The three-step process of cellulose biosynthesis was represented within the EUCAWOOD unigenes set [49,50]. Three sucrose synthases (SuSy) were found: one was similar to *At*SUS1 whereas the other two were similar to *At*SUS4 [51]. In addition, five unigenes homologous to members of the cellulose synthase (*CesA*) multigene family were also found that correspond to the *EgCesA1*-*EgCesA5 *genes recently described in *E. grandis *[52]. *EgCesA1, EgCesA2 and EgCesA3 *are specifically expressed during secondary cell wall biosynthesis, whereas expression of *EgCesA4 *and *EgCesA5 *is linked to the synthesis of primary cell wall. Two unigenes similar to *KORRIGAN (KOR) *proteins were alsoretrieved from EUCAWOOD. Several studies have proven the importance of KOR proteins in the formation of the plant cell wall in various species. For instance, *Arabidopsis irx2 *and *kor1 *mutations, which map to the same gene, both affect secondary growth [53].

The EUCAWOOD set also contained unigenes with homologies to *Arabidopsis *proteins dedicated to hemicellulose and pectin biosynthesis including three putative cellulose synthase-like genes, thought to be involved in the synthesis of the backbone structures of mannans, glucomannans and galactomannans [54]. We also found eight unigenes similar to UDP-xylose synthases, one to UDP-xylose epimerase, two to β-xylosidases, one to glucuronic acid epimerase, two to pectin esterases, four to pectate lyases and four to polygalacturonases.

Several unigenes similar to other gene families thought to be involved in cell wall formation were also found. Two unigenes were similar to *PttGH19A*, which encodes a chitinase-like protein highly expressed during poplar secondary cell wall biosynthesis [55]. Mutation of two genes similar to *PttGH19A *in *Arabidopsis (At1g05850 *and *At3g16920*) caused deficient biosynthesis and incorporation of cellulose into the cell wall, as well as ectopic lignin deposition and aberrant cell shapes with incomplete cell walls [56].

Genes encoding proteins involved in loosening and rearrangement of the cell wall were also present among the EUCAWOOD unigenes, including, for instance, two expansin genes. Expansins are thought to directly promote cell expansion by hydrolysing noncovalent bonds between cellulose and hemicelluloses in the cell wall [57]. The action of expansins is facilitated by xyloglucan endotransglycosylases (XETs)/hydrolases (XEHs), also known as XTHs, which incorporate and modify xyloglucans into the cell wall [58]. XTH proteins are members of the glycosyl hydrolase (GH) family 16, which is the most abundant carbohydrate-metabolising enzyme group among the EUCAWOOD matches in the CWN database, represented by 19 unigenes. A total of 41 gene models belonging to the GH16 family have been recorded in the genome of poplar [54].

Whereas carbohydrates and lignin constitute the bulk of cell wall materials, structural proteins also form a network that contributes to the architecture and functionality of the cell wall. This is the case for fasciclin-like proteins (FLA), a subgroup of arabinogalactan proteins involved in processes such as growth and cell proliferation. Five FLAs were identified in the EUCAWOOD unigene set. All five are similar to *AtFLA11 *and *AtFLA12*, whose expression is linked to secondary cell wall biosynthesis and maturation [59].

### Transcription factors

Given the importance of transcriptional regulation during wood formation, we carried out BlastX searches comparing the EUCAWOOD unigene set with the Plant Transcription Factor Database (PTFD) [60] and the Database of *Arabidopsis *Transcription Factors (DATF) [61]. A total of 141 unigenes (110 Cg and 31 Sg) had at least one hit in either database. PTFD and DATF produced 136 and 103 hits respectively, with 98 unigenes having a hit in both databases (Additional file 8). Interestingly, 90 of the 136 PTFD hits corresponded to poplar sequences, whereas only 24 matched *Arabidopsis *and 10 matched rice proteins. The 141 hits identified 41 transcription factor families, some of which are known to play a role in secondary growth and wood formation [8,62]. The 'C2H2 zinc-finger' family was the most frequently represented among the EUCAWOOD unigenes, with 15 putative members, followed by the MYB and NAC families, each represented by 11 putative unigenes. A number of plant MYB proteins, including *Eucalyptus *and other woody species, have already been proven to regulate the biosynthesis of phenolic compounds, including lignin [22,23,62,63]. Putative orthologs of NAC factors known to play a role in xylem differentiation were found among the EUCAWOOD sequences. For instance, the NAC secondary wall thickening promoting factor genes *NST1 *and *NST3 *are implicated in the formation and thickening of secondary wall in *Arabidopsis *[64,65]; *ANAC012/SND1*, a member of the IIb group of the *NAC *family, has recently been described as a key regulator of xylary fiber development [66,67]. A putative ortholog of the negative regulator of both secondary cell wall synthesis and programmed cell death, *ANAC104/XND1 *[68], was also present in EUCAWOOD. Three unigenes resemble LIM transcription factors, some of which have been shown to regulate the expression of lignin biosynthetic genes [69,70]. In fact, Cg2892 is similar to *EcLIM1 *from *E. camaldulensis*, which shares 86% homology with *Nicotiana tabacum NtLIM1*. Suppression of *NtLIM1*expression caused the downregulation of lignin biosynthesis genes such as phenylalanine ammonia-lyase (*PAL*), 4-coumarate CoA ligase (*4CL*), cinnamate 4-hydroxylase (*C4H*), and cinnamyl alcohol dehydrogenase (*CAD*) [69,70].

The auxin-inducible factor (AUX/IAA) and auxin-response factor (ARF) families were represented by four EUCAWOOD unigenes. One is similar to *IAA13 *and its closely related *BDL/IAA12*, whose mutation disrupts the normal cell and tissue organization along the apical-basal axis resulting in discontinuous and reduced vascular formation [71].

Six homedomain-leucine zipper proteins were present in the EUCAWOOD dataset. Among them, one contig (Cg3498) is similar to *ATHB15 *and *ATHB8*, members of class III (HD-ZIPIII). These proteins are involved in vascular development and wood formation and share antagonistic functions with other HD-ZIPIII proteins such as REVOLUTA, PHABULOSA (PHB), and PHAVOLUTA [72]. A putative ortholog of PHB, known to positively regulate the size of the vascular bundles, was also found in the EUCAWOOD set [72].

### Core xylem genes

Expression profiling has been used in several studies to report sets of genes differentially expressed during xylem development, notably in arabidopsis [46,73,74]. Comparison of the EUCAWOOD unigenes with sets of genes expressed during xylem differentiation in arabidopsis, revealed four candidate genes common to all the above-mentioned studies. They encode IRX9 (At2g37090; a GT family 43), COBL4/IRX6 (At5g15630; a COBRA-like protein), IRX8 (At5g54690; a GT family 8) as well as a protein of unknown function (At4g27435). These four genes belong to a group of 52 arabidopsis genes defined by Ko and collaborators as 'core xylem-specific genes' in their comparative transcriptome analysis [74].

### *In silico *identification of simple sequence repeat (SSR) markers

Genomic SSR markers or microsatellites have already been developed in *Eucalyptus *species [75,76], however, to the best of our knowledge, only one very recent paper was dedicated to EST-SSRs [77]. To mine the EUCAWOOD dataset for EST-SSRs, we looked for di- and tri-nucleotide repeats stretching for at least 12 nt and also tetra- to hexa-nucleotides repeated at least three times. A total of 639 putative microsatellites were thus found in 512 EUCAWOOD unigenes (Additional file 9). That is, 13.3% of the EUCAWOOD unigenes contain at least one putative SSR. This agrees with the frequency of SSR-ESTs found in other dicotyledonous species, which ranges from 2.65–16.82% [78].

Tri-nucleotide repeats (TNRs) were the most abundant motifs (46.3% of the total 639 SSRs), followed by di-nucleotide repeats (DNRs, 29.4%). This is consistent with most similar studies of monocots as well as dicots [78,79]. Among the TNRs, the most abundant motifs were AAG/AGA/GAA/CTT/TTC/TCT (96 EST-SSRs) representing 32.3% of TNRs and 14.9% of all SSRs. The DNR the most represented was AG/GA/CT/TC (165 EST-SSRs), which accounted for 87.8% of all DNRs and 25.9% of all SSRs. These motifs have also been found to be the predominant DNRs and TNRs among the EST-SSRs in more than 20 plant species [78,79].

### The EUCAWOOD database

EUCAWOOD [26] is a MySQL database allowing four types of queries through a web interface consisting of check boxes and pull-down menus. Query 1 is a library filter query allowing retrieval of all unigenes or a selection of them from the user-specified libraries. EST assembly, Blast hits against several databases (Uniref 100, CWN, MAIZEWALL ...), GO and PFAM annotations can also be retrieved. Query 2 retrieves unigenes by name (aliases), key words, PFAM or GO annotations, or hits in Blast (accession number or name). Query 3 allows Blast searches (blastn, tblastx, tblastn) for a user-specified sequence (or batch of sequences) in the EUCAWOOD database. Query 4 gives access to a tree view showing the number of unigenes by GO terms.

## Conclusion

We report the sequencing, assembly and annotation of approximately 10,000 ESTs derived from a normalised full-length secondary xylem cDNA library as well as subtractive libraries. Our data demonstrate the benefit for large-scale gene/EST discovery of using normalized libraries that minimize redundancies and increase the representation of the different genes expressed in a chosen tissue. They also illustrate the advantage of sequencing, in parallel, ESTs from subtracted libraries, which are enriched in clones not found in cDNA libraries and are a valuable source of new genes. The combination of a normalised secondary xylem library and subtractive libraries allowed us to assemble a large set of wood-related *Eucalyptus *unigenes, called EUCAWOOD, thus substantially increasing the representation of *Eucalyptus *ESTs available in public databases. The number of sequences available for this economically important genus has increased significantly during the past months [80-82] but is still low in comparison to other forest tree species such as poplar or pine. The major part of this new data set is composed of short sequences whose number is expected to increase dramatically in the future thanks to the development of the high-throughput '454' technology [81].

The EUCAWOOD dataset currently provides the most comprehensive list of unigenes dedicated to wood formation in the genus *Eucalyptus*. We have provided a public database supporting multiple queries that will be a particularly valuable resource for the correct annotation of genomic sequences and for the functional analysis of genes and their products. The most immediate application of the EUCAWOOD unigene set reported in this study is the development of a wood reference microarray for *Eucalyptus*.

Finally, the EUCAWOOD dataset is also a valuable source of microsatellite markers as 639 EST-SSRs were identified from it. The usefulness of these EST-derived SSRs is superior to that of the genomic SSRs especially in looking for markers for important traits using the Gene Candidate approach. They are also usually more conserved and, therefore, may be easily transferred between species. The microsatellites reported for all these unigenes might be used to produce genetic maps, providing resources, for instance, for trait/gene association and candidate gene identification for wood quality traits.

## Methods

### Normalization of a *Eucalyptus *secondary xylem cDNA library

A library of directionally-cloned cDNAs prepared from the developing secondary xylem tissue of *Eucalyptus gunnii *was constructed in the λ ZapII vector (Stratagene, Amsterdam, The Netherlands) [17]. The library normalization process was based on the reassociation of an excess of cDNA inserts (driver DNA) to the cDNA library in the form of single-stranded circles (tracer DNA) as described by Bonaldo et al. [24]. A pBluescript SK vector carrying a *Homo sapiens *desmin cDNA (accession N° BC032116) was added at 1,000 copies to the initial library in order to assess the normalization efficiency. Single-stranded pBluescript phagemid DNA was generated *in vivo *from approximately 1.5 × 10^6 ^library clones and purified by hydroxyapatite (HAP) chromatography. Double-stranded driver DNA was generated by PCR from 1 ng of single-stranded library plasmid DNA with SK and T7 primers flanking the pBluescript vector multicloning site. PCR products were purified on a Qiagen Spin Column PCR Purification kit (Qiagen, Courtaboeuf, France) and eluted in TE buffer. Hybridization was performed by mixing 250 ng of single-stranded library phagemids with an excess of the PCR-amplified driver DNA and of each 3', 5' and oligo d(T20) blocking oligonucleotides. Hybridization was performed at 30°C for 24 h (Co*t *= 5). Single-stranded phagemids were purified by using HAP chromatography and converted to double strands by using SEQUENASE v2.0 DNA polymerase (USB, Staufen, Germany) and M13 primer. Double-stranded plasmids were electroporated into *Escherichia coli *DH10B cells (Invitrogen, Cergy Pontoise, France) and transformed cells were selected by growth on ampicillin.

### Preparation of juvenile and mature secondary xylem RNAs

Juvenile and mature secondary xylem samples were harvested from four-year-old and 10-year-old trees, respectively. Samples were collected from trees of a single *Eucalyptus globulus *genotype (clone vc9, RAIZ, Portugal). Tissue collection and RNA extraction were performed as described by Southerton et al. [83]. Remaining traces of DNA were removed with RQ1-RNAase-free DNAase (Promega, Madison, WI, USA) according to the manufacturer's procedure. RNA quality was checked by both agarose gel electrophoresis and spectrophotometry.

### Construction and normalization of EST subtractive libraries

The secondary xylem subtractive libraries were constructed by using the SSH technique [25]. SSH was performed with the PCR-Select cDNA Subtraction kit (Clontech Laboratories, Mountain View, CA, USA), according to the manufacturer's procedure. The subtracted PCR products generated by SSH were inserted into pGEM-T Easy Vector (Promega) and cloned into *E. coli *DH5α. Clones of recombinant bacteria were tested for complementation [84]. White colonies were picked with a BioPick robot (Genomic Solutions, Huntingdon, Cambridgeshire, UK) and arrayed in 384-well plates containing ampicillin (100 μg/ml)-supplemented LB freezing medium (25 g/l LB broth, 6.3 g/l K_2_HPO_4_, 1.8 g/l KH_2_PO_4_, 0.5 g/l sodium citrate, 1 g/l MgSO_4_, 0.9 g/l ammonium sulfate, 4.4% glycerol). All recombinant clones were grown at 37°C overnight then stored at -80°C. High-density colony arrays (HDCA) were produced, hybridized and analyzed in order to eliminate false-positive clones. For this purpose all bacterial clones were spotted onto nylon membranes and hybridized with labeled SMART cDNAs from two independent juvenile and mature xylem probes as previously described [14,15]. ANOVA was performed on normalized data enabling us to keep 818 clones showing a significant relative expression level change (ratio of 1.2) between the two developmental stages.

### Data processing and assembly

Sequencing of the *Eucalyptus *secondary xylem cDNA library and SSH libraries was done at the Genoscope facilities (Centre National de Séquençage, Evry, France). Crossmatch software [85] was used to trim vector from the sequences. Subsequently, a home-made script was run to detect chimeras and remove low quality sequences. Sequences longer than 50 nucleotides and with a 'phred20 score' in at least 80% of the sequence were selected as good quality sequences suitable for assembly. The presence of poly A and poly T in the middle of the sequence was regarded as an indication of a chimeric sequence, which was then split in two and treated as two independent sequences. Good quality sequences were submitted to EMBL or GenBank according to the database curators' instructions.

Publicly available *Eucalyptus *ESTs and mRNA sequences were downloaded from the GenBank database at the NCBI server using the Entrez tool in March 2008. Wood-related ESTs produced in our lab were eliminated to avoid redundancy. The FASTA files containing good quality wood-related sequences and the GenBank *Eucalyptus *sequences were combined and then assembled with CAP3 software [86] using default parameters.

### Sequence annotation

The NCBI BLAST program version 2.2.6 was used to perform BLASTX similarity searches of various protein databases: UniRef100 was downloaded from the EBI ftp site [31] in March 2008; TAIR7 peptides were downloaded from The *Arabidopsis *Information Resource web site [28]; PoplarProteins1.1b JamboreeModels were downloaded from the Joint Genome Institute *Populus trichocarpa *v1.1 web site [29]; rice sequences were downloaded from the TIGR Rice Genome Annotation website [30]. The expectation (E)-value threshold used for BlastX searches was 10^-10^.

For functional characterization purposes, the Gene Ontology (GO) terms associated with the Uniref100 database were downloaded from the ftp site at the EBI [87]. The GO terms allocated to the best Uniref100 hit were assigned to the corresponding unigene. BLASTX searches (E value ≤ 10^-10^) were likewise conducted against the Cell Wall Navigator Database [38,39], the Maizewall database [40], the Plant Transcription Factor Database [60] and the Database of *Arabidopsis *Transcription Factors [61].

### Data storage

A MySQL database was developed to store the raw ESTs, the good quality ESTs, the assembly results (contigs and singletons), the BlastX results as well as the GO and PFAM annotations [26]. Programs written in PHP and PERL languages were developed to load and export the data. The reports, figures and tables were built by querying the database using the SQL query language.

### *In silico *identification of simple sequence repeat (SSR) markers

The MREPS software [88] was used to identify and localize the microsatellite motifs in the assembled EUCAWOOD unigene set. We looked for di- and tri-nucleotide repeats stretching for at least 12 nucleotides and also tetra- to hexa-nucleotides repeated at least three times.

## List of the abbreviations used

ABC: ATP-binding cassette; CesA: cellulose synthase; Cg: contig; CWN: Cell Wall Navigator database; DATF: Database of *Arabidopsis *Transcription Factors; DNR: di-nucleotide repeat; EST: expressed sequence tag; FLA: fasciclin-like arabinogalactan protein; GH: glycosyl hydrolase; GO: gene ontology; GT: glycosyl transferase; HCT: hydroxycinnamoyl-CoA:shikinimate/quinate hydroxycinnamoyltransferase; ID: identifier; Jm: *Eucalyptus globulus *juvenile vs mature secondary xylem SSH library; Mbp: million base pairs; Mj: *Eucalyptus globulus *mature vs juvenile secondary xylem SSH library; nt: nucleotide; PTFD: Plant Transcription Factor Database; Sg: singleton; SSH: suppression subtractive hybridisation; SSR: simple sequence repeat; SuSy: sucrose synthase; TNR: tri-nucleotide repeat; XET: xyloglucan endotransglycosylases; Xl: *Eucalyptus gunnii *secondary xylem *vs *mature leaf SSH library; Xp: *Eucalyptus gunnii *secondary xylem *vs *secondary phloem SSH library; Xyl_cDNA_: *Eucalyptus gunnii *secondary xylem full-length cDNA library.

## Competing interests

The authors declare that they have no competing interests.

## Authors' contributions

DR designed bioinformatics approaches, analyzed the sequence and bioinformatics data, sorted the M_j _and J_m _SSH libraries and wrote the manuscript; HSC designed and created the EUCAWOOD database and its interface; HSC and FS designed bioinformatics approaches and provided bioinformatics tools, pipelines and scripts; NL normalized the Xyl_cDNA _library and participated in managing and sequencing all the libraries described in the manuscript; EP built the M_j _and J_m _SSH libraries and contributed to discussions on the manuscript; PW and AC sequenced the libraries; PS and JGP contributed to writing the manuscript as well to discussions on its content; JGP supervised and coordinated the project. All authors read and approved the manuscript.

## Supplementary Material

Additional file 1 **EUCAWOOD unigenes annotation**. Data concerning all the 3,857 EUCAWOOD unigenes are shown, including: the wood-related ESTs in every unigene; the best BlastX hit for each unigene (E value ≤ e^-10^) in all the searched protein databases (*i.e. *Uniref100, JGI poplar proteins v1.1, *Arabidopsis *TAIR7 peptides, TIGR Rice genome annotation, NCBI grapevine), as well as the GO and PFAM annotation terms and IDs allocated to the first Uniref100 hit of each unigene. Click here for file

Additional file 2 **EUCAWOOD unigenes matching poplar and/or grapevine but not *Arabidopsis *or rice sequences**. Lists of EUCAWOOD unigenes matching poplar, grapevine or both, but not *Arabidopsis *or rice sequences. Click here for file

Additional file 3 **GO terms and ID assignments to EUCAWOOD unigenes**. GO terms and PFAM ID assignments to EUCAWOOD unigenes. Click here for file

Additional file 4 **PFAM assignments for EUCAWOOD unigenes. Comparison of Jm and Mj libraries**. PFAM assignments for EUCAWOOD unigenes. Comparison of Jm and Mj libraries. Click here for file

Additional file 5 **EUCAWOOD 'No Hits'**. Unigenes are shown that have no matches in the Uniref100, poplar, grapevine, *Arabidopsis *or rice protein databases. Click here for file

Additional file 6 **Identification of cell wall related genes in EUCAWOOD**. EUCAWOOD unigenes presenting BLASTX hits against the Cell Wall Navigator [38,39] and/or MAIZEWALL [40]databases are shown with the best hit in either database (E value ≤ e^-10^). Click here for file

Additional file 7 **EUCAWOOD lignification toolbox**. EUCAWOOD unigenes were mined for homologous genes in the *Arabidopsis *lignification toolbox [41]. The table shows the best hit of each unigene against Uniref100 and TAIR7 Peptides databases. For every hit, the corresponding alignment score (E value ≤ e^-10^) is shown. Click here for file

Additional file 8 **Transcription factors among EUCAWOOD unigenes**. EUCAWOOD unigenes with BLASTX hits in the *Arabidopsis *Transcription Factor database (TFD) or in the Plant Transcription Factors database (PDFD) are shown, along with the best hit in either database, and the corresponding alignment score (E value ≤ e^-10^). Click here for file

Additional file 9 **EUCAWOOD putative SSRs or microsatellites**. *In silico *identification of simple sequence repeat (SSR) markers in the EUCAWOOD database. Click here for file
